# Detection and imaging of lipids of *Scenedesmus obliquus* based on confocal Raman microspectroscopy

**DOI:** 10.1186/s13068-017-0977-8

**Published:** 2017-12-13

**Authors:** Yongni Shao, Hui Fang, Hong Zhou, Qi Wang, Yiming Zhu, Yong He

**Affiliations:** 1 Shanghai Key Lab of Modern Optical System, University of Shanghai for Science and Technology, No. 516, Jungong Road, Shanghai, 200093 China; 2 College of Biosystems Engineering and Food Science, Zhejiang University, Hangzhou, 310058 China

**Keywords:** Raman microspectroscopy, Microalgae, Lipids

## Abstract

In this study, confocal Raman microspectroscopy was used to detect lipids in microalgae rapidly and non-destructively. Microalgae cells were cultured under nitrogen deficiency. The accumulation of lipids in *Scenedesmus obliquus* was observed by Nile red staining, and the total amount of lipids accumulated in the cells was measured by gravimetric method. The signals from different microalgae cells were collected by confocal Raman microspectroscopy to establish a prediction model of intracellular lipid content, and surface scanning signals for drawing pseudo color images of lipids distribution. The images can show the location of pyrenoid and lipid accumulation in cells. Analyze Raman spectrum data and build PCA-LDA model using four different bands (full bands, pigments, lipids, and mixed features). Models of full bands or pigment characteristic bands were capable of identifying *S. obliquus* cells under different nitrogen stress culture time. The prediction accuracy of model of lipid characteristic bands is relatively low. The correlation between the fatty acid content measured by the gravimetric method and the integral Raman intensity of the oil characteristic peak (1445 cm^−1^) measured by Raman spectroscopy was analyzed. There was significant correlation (*R* ^2^ = 0.83), which means that Raman spectroscopy is applicable to semi-quantitative detection of microalgal lipid content.

## Background

The world energy crisis is intensified continuously nowadays, which raises interest in looking for renewable energy resources. Among the existing renewable energy sources, biodiesel is the most widely used variety which also have the fastest speed of development. Microalgae have good potential for fuel production because of their promising biomass feedstock and ability to synthesize large amounts of certain chemical compounds from sunlight and carbon dioxide [1, 2]. Under the environment of nitrogen stress, some kind of microalgae (e.g., *Chlorella pyrenoidosa*, *Scenedesmus obliquus*…) significantly synthesize and accumulate lipids, mainly in the form of triacylglycerol (TAG) [3, 4].

Several methodologies used for quantitative analysis of microalgae lipids, such as gas chromatography–mass spectrometry (GC–MS), are not just time-consuming and ruinous, but also requires sophisticated sample preparation process which produces hazardous chemical waste. Although GC–MS provide precise analysis of lipid composition, it cannot provide the detailed information of lipid metabolism. Vital staining methods using Nile Red or BODIPY 505/515 can show lipid distribution in a single cell but cannot provide some desired lipid characteristics such as chain length and degree of unsaturation [5, 6]. Studies showed that the lipid content of one cell was significantly correlated with the fluorescence intensity of Nile Red combined with the lipid inside the cell [7, 8].

Confocal Raman microscopy is a powerful tool for physicochemical characterization of biological samples, which directly detects vibrations of biochemical bonds through the inelastic scattering by a laser light [9]. It enables single-cell, in vivo monitoring of various cellular components in a rapid, non-destructive, label-free and quantitative manner [10–13]. With the assistance of chemometric methods, Raman spectroscopy can be applied to wide research area like microalgae species identification [14], water pollution identification, and nutritional status identification [15–17]. A study showed that the water bodies under different nitrogen nutrition conditions can be effectively identified by the LDA classification model established based on lipid-related Raman shift [18]. Heraud described a in vivo method for predicting the nutrient status of individual algae cells using Raman microscopy and partial least squares discriminant analysis [19]. Samek employed the characteristic peaks in the Raman scattering spectra at 1656 and 1445 cm^−1^ as the markers defining the ratio of unsaturated-to-saturated carbon–carbon bonds of the fatty acids in the algal lipids [20]. In situ and in vivo chemical compounds distribution and concentration can be shown by Raman spectrum data processing [21–24].

At the present time, the research of microalgae lipid production in China is mostly focused on promoting the ability of lipid production of microalgae through cultivation environment modifying or genetic engineering as well as optimization of lipid conversion technology. Development of rapid and non-destructive Raman spectrum testing process toward microalgae lipid will accelerate the research on biodiesel production. Problems still remain in the direct quantitative and qualitative detection research of microalgae lipid using Raman spectrum. In our preliminary experiments, we found that the characteristic peaks of lipids are not obvious due to the concealment effect of pigments (e.g., chlorophyll, carotenoids). The object of the preliminary experiment was *Chlorella* sp. Although this kind of microalgae can accumulate large amounts of lipids, its cellular volume is so small that obstructs the acquisition of spectrum and the process of data. The object of this study was a different kind of microalgae, *S. obliquus*. *Scenedesmus obliquus* is a Chlorophyta that the dry weight of its cellular lipid content can be accounted for more than 50% of the weight of cells. The large cell individual is suitable for the detection research of Raman point and surface scan. Under normal growth conditions, the cells of *S. obliquus* are fusiform. Combined living form of four cells is common, which can be changed under the stress environment.

This study, using *S. obliquus* as the experimental subject, verified the ability of Raman spectroscopy to classify cells collected from different days cultivated under nitrogen stress, and discussed the optimization of detection process according to the Raman mapping measurement imaging results of single cells.

We first list the materials and methods of the experiment, including the cultivation of microalgae, Nile Red staining and observing process, gravimetric analysis of total lipid content, GC–MS analysis of lipid composition and Raman spectral acquisition. We next build a PCA-LDA model to classify microalgae cells collected from different days, and its correct classification rate reached to 100%. We then make visual analysis of cellular lipids and pigments and cell growth pattern based on the Raman mapping data. The paper also attempts to discuss the correlations between the lipid content measured by gravimetric analysis and Raman mapping measurement.

## Methods

### Algae species and culture conditions

*Scenedesmus obliquus*, FACHB-276, was purchased in Freshwater Algae Culture Collection at the Institute of Hydrobiology, FACHB-collection. The SE basal medium was configured according to the standards provided by the Institute of Hydrobiology. *Scenedesmus obliquus* was expanded in the SE medium for 30 days and cultured to a stable growth stage. 2 l BG11 nitrogen-deficient (BG11-N) medium was configured with NaNO_3_ concentration of 0.5 g/l. 1 l uniform algae fluid was centrifugated and then liquid supernatant was discarded. The washed algal mud was quickly added to the BG11-N medium, placed at a constant temperature (25–27 °C), under uniform illumination (2000–3000 lx), and under periodic illumination conditions (12 l:12 days) cultured continuously for 9 days. Before each experiment, the microalgae cell concentration was estimated by the cell count plate method to ensure that the microalgae were in stable growth during the whole experiment.

### Intracellular lipid accumulation observation

The intracellular lipid of *S. obliquus* was observed by Nile red staining. 1.5 ml uniform algae fluid and 0.5 ml DMSO were mixed, and oscillated 10 min for breaking cytoderm. 20 μl NR liquor (0.1 g/l) was then added, and oscillated 10 min for dyeing evenly. A 2 μl stained algae fluid was taken for the observation by fluorescence microscope (Nikon Eclipse 90i, 20× objective lens). The excitation wavelength range is 505–566 nm. Pictures of the stained algae cells were taken using corresponding image processing software.

### Determination of total lipid content

The total fat content was determined by gravimetric method. 50-ml centrifuge tube (M0) was washed and weighed after drying. 30 ml uniform algae fluid was added in the centrifuge tube, and liquid supernatant was discarded after centrifugation. The algae mod was dried in the centrifuge tube in the drying oven under 60 °C then weighed (M1). Some 1 mol/l HCL was added and then heated the mixture in 80 °C water bath for 30 min: liquid supernatant was discarded after centrifugation. The extraction methods were performed as per the Bligh–Dyer method [25]. 6 ml methanol:chloroform = 2:1 mixed solution was added into the residual, and oscillated by the vortex oscillator until all the algal residue turned white. After 4-h stewing, the chloroform layer was transferred to a culture dish that had been washed, dried and weighed (M2). It was dried to constant weighing in the draught cupboard then weighed (M3). Lipid content (LC) was calculated according to the following formula:

1 $${\text{LC}}\,\left( \% \right) = \frac{M3 - M2}{M1 - M0}.$$

### Determination of lipid composition

The oil composition of algae cells was directly treated with methyl esterification. Fatty acid methyl ester (FAMEs) was analyzed by gas chromatograph-mass spectrometer (GC–MS). At ninth day, 240 ml uniform algae fluid was centrifugated and the supernatant was discarded. The algae mud was transferred to a 50-ml round-bottomed flask and crashed it using ultrasonication for 10 min. 10 ml reactant mixture (methanol:chloroform: HCL = 10:1:1) was added into the algae mud, mixed, and then refluxed in 90 °C water baths for 4 h. After the reaction is complete, the flask was removed and cooled it to room temperature. 3 ml hexane was added into the mixture for extracting the FAMEs, and repeated three times. The extract was dried with nitrogen and then added with 1 ml hexane. 37 FAMEs mixture standard heating process was used for GC–MS detection.

### Raman spectrum acquisition

#### Sample preparation

1.5 g powdered agar and 50 ml distilled water were mixed, boiled for 2 min, and then cooled to 40 °C. 2 ml algae fluid was transferred to a clean centrifuge tube, and 2 ml prepared agar solution was added. The mixture was cooled and solidified The solidified sample was cut into thin slices and was placed it on the slide.

#### Data acquisition

The Renishaw laser confocal micro Raman spectrometer (Renishaw PLC, United Kingdom/InVia–Reflex 532/XYZ) was the main instruments for this study. Data acquisition software WiRE3.3 was used to adjust the acquisition parameters. Specific parameters were as follows: the excitation wavelength is 532 nm; the spectral collection range is 633–1813 Raman shift/cm^−1^; the laser intensity is 1.5 W; and the exposure time is 2 s. 15 single data were acquired . *S. obliquus* cells were selected with different growth forms and mapping data were acquired, after the target area was selected; acquisition step is 0.5 μm. 1–2 sets of mapping data were acquired for each growth morphology of the cells. The experiment was performed every other day; 75 sets of single data and 20 sets of mapping data were acquired altogether.

### Spectral data processing

#### Single data processing and modeling

In this paper, we use Baseline correction, Savitzky–Golay smoothing (SG) and data normalization to preprocess single data. The purpose of the baseline correction is to eliminate the baseline drift caused by the instrument or other interference and reduce the data error through polynomial fitting and other mathematical algorithms [26]. Through the least square fitting of spectral data, SG can reduce data noise level and retain the characteristics of spectral distribution, such as characteristic peak height, relative maximum, minimum and spectral peak width to a great extent [27]. Different spectral data processings apply different data normalization formulas. In this paper, the relative content of oil and chlorophyll is used to describe the accumulation of oil over time.

In order to reduce modeling variables and computation, principal components analysis (PCA) was used to reduce the dimension of spectral data after pretreatment [28]. Linear discriminant analysis (LDA) was used for modeling. LDA is a classical algorithm for pattern recognition. The purpose is to obtain the best separability of the model in the space by vector projection [29]. Data processing softwares were MATLAB R2015b and The Unscrambler X^®^.

#### Mapping data processing

Baseline correction and SG smoothing were used for preprocessing the mapping data. After selecting the peak of target characteristic, the pseudo color image of lipids distribution is drawn by interpolation method.

## Results and discussion

### Content and composition of lipids in *S. obliquus*

**Fig. 1:**
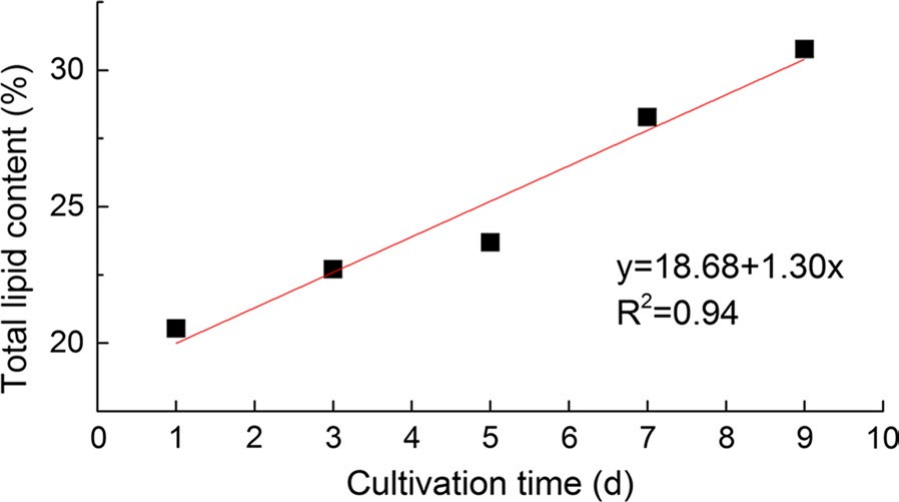
Fitting curve of total lipid content of *S. obliquus*

Under nitrogen stress, *S. obliquus* showed significant accumulation and accumulation of lipids. The total lipid content of microalgae was 20.54% before stress culture; this value increased to 30.77% at the ninth day of stress culture (Fig. 1). Rate of increase was 49.80%.

The GC/MS result (Table 1) shows that saturated fatty acids (SFA) and unsaturated fatty acids (UFA) synthesized by *S. obliquus* cells accounted for 55.66 and 44.34%, respectively. Monounsaturated fatty acids (MUFA) and polyunsaturated fatty acids (PUFA) accounted for 14.46 and 29.89%, respectively. In addition, small amount of docosahexaenoic acid (DHA) and eicosapentaenoic acid (EPA) were detected. As for the number of fatty acids, 47.83% of the accumulated oil in this algae species under nitrogen deficiency environment is odd carbon fatty acid (OCFA). Microorganisms tend to synthesize high ratio of OCFA, because of its unique metabolic process different from higher animals and plants [30]. The above analysis results show that, with the capability of synthesizing large number of long-chain fatty acids, *S. obliquus* can be used as potential algae for biodiesel production. UFA synthesized by these algae has nutrient value; OCFA has industrial and health care value [31].

**Table 1:** Fatty acid composition of *S. obliquus*

Fatty acids	Content/%
C14:0	1.42
C15:0	35.32
C16:0	4.77
C16:1	4.20
C16:3	1.94
C17:0	10.30
C18:0	3.84
C18:1	8.05
C18:2	1.71
C18:3	25.12
C19:1	2.21
C20:5 (EPA)	0.40
C22:6 (DHA)	0.72
SFA	55.66
UFA	44.34
MUFA	14.46
PUFA	29.89
OCFA	47.83

### NR microscopic examination of *S. obliquus*

**Fig. 2:**
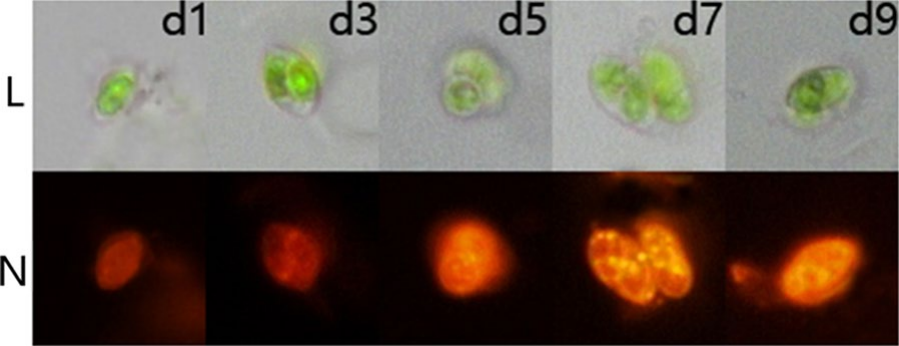
Light-field images (*L*) and NR fluorescent images (*N*) of *S. obliquus* at different days after lipid accumulation induction

The results of NR staining microscopy (Fig. 2) showed that the lipid content of *S. obliquus* cells accumulated obviously during the period of nitrogen stress. Most of the cells were spindle shaped, and the pyrenoid in the cells was clearly visible. During the cultivation period, size and intracellular chlorophyll content of *S. obliquus* cells had not changed. Because of the polar lipids on the surface of the algal cells, the color of cells in the fluorescent images was basically orange, and the region of pyrenoid was also orange. Bright yellow spots appeared over time, which were the lipid particles that were accumulated in the algae cells.

### Visual analysis of intracellular constituents in *S. obliquus*

**Fig. 3:**
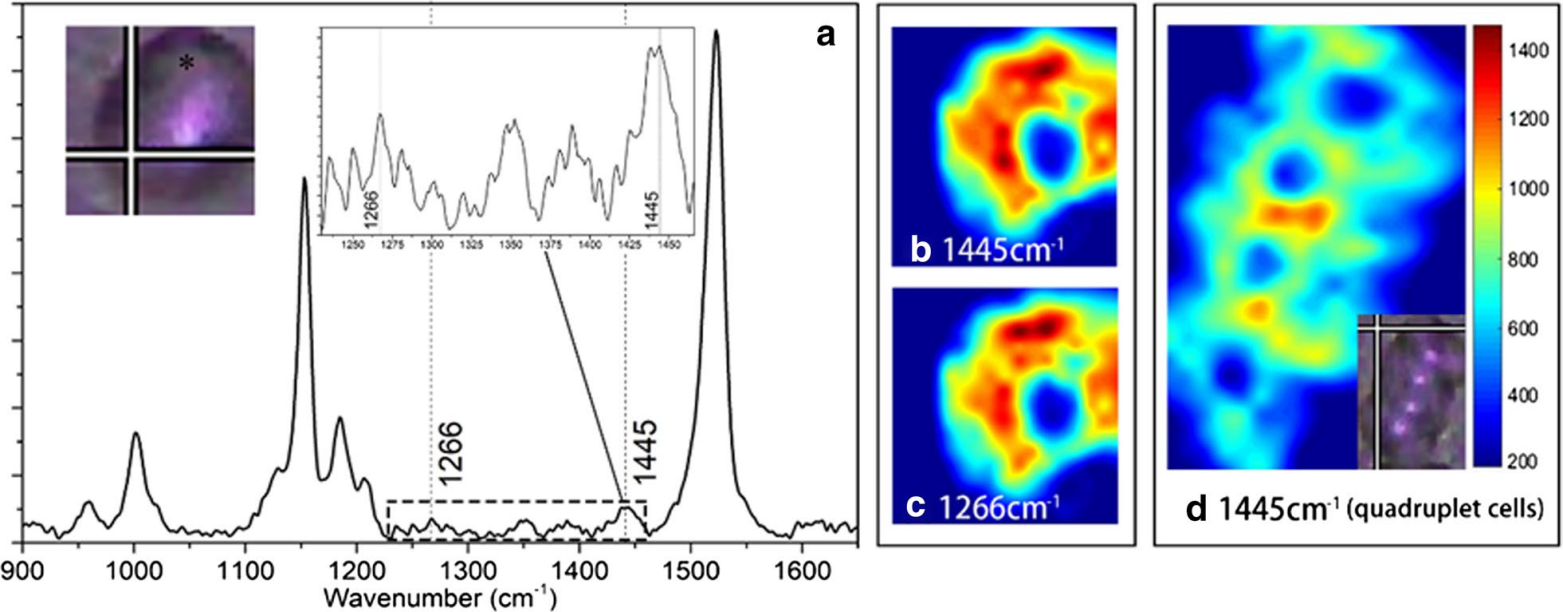
Raman image obtain of *S. obliquus*. **a** Raman spectra after pretreatment locate at the black asterisk in the inset image. The left inset image shows the bright-field image of a single cell of *S. obliquus*, and the other inset image shows partial enlarged detail of lipid characteristic peaks. **b**, **c** Raman images of the same cell at **b** 1445 cm^−1^, **c** 1266 cm^−1^. **d** Raman image and bright-field image of quadruplet cells of *S. obliquus* at 1445 cm^−1^

In order to assess the ability of Raman spectroscopy to detect the intracellular substances of *S. obliquus*, visualization processing of Raman mapping data was carried out. In Raman spectrum of *S. obliquus* cells, the peaks of 1266 and 1656 cm^−1^ corresponded to the cis = C–H in-plane deformation and the cis C = C stretch, respectively. These two peaks mainly represented the degree of unsaturation of the fat chain. The peaks of 1302 and 1445 cm^−1^ corresponded to the CH_2_ twist and the CH_2_ bend, respectively. These two peaks were assigned to saturated carbon chain [32]. Raman images of saturated fatty acid (1445 cm^−1^) and unsaturated fatty acid (1266 cm^−1^) were constructed at the ninth day of nitrogen stress (Fig. 3b–d). A typical Raman spectrum of *S. obliquus* cell is shown in Fig. 3a. There was no obvious Raman response in a circular region in the Raman images of algal cells, indicating that there was no accumulation of neutral lipids in these areas, which should be the region of pyrenoid. This result was consistent with the results of NR microscopy. In single algae cells, the Raman response high value is dotted, corresponding to the accumulation of lipid particles in the cells. The distribution of saturated fatty acids is slightly different from the distribution of unsaturated fatty acids. *Scenedesmus obliquus* often grow in four-cell combination form. Figure 3d displays a Raman image of four-cell combination form of *S. obliquus*, of which four pyrenoids are obvious. The overall Raman response of four-cell combination was significantly lower than that of single cell, indicating that lipid accumulation level was relatively low, probably due to the combined growth form which is not conducive to lipid accumulation.

### PCA-LDA modeling based on Raman spectroscopy

**Fig. 4:**
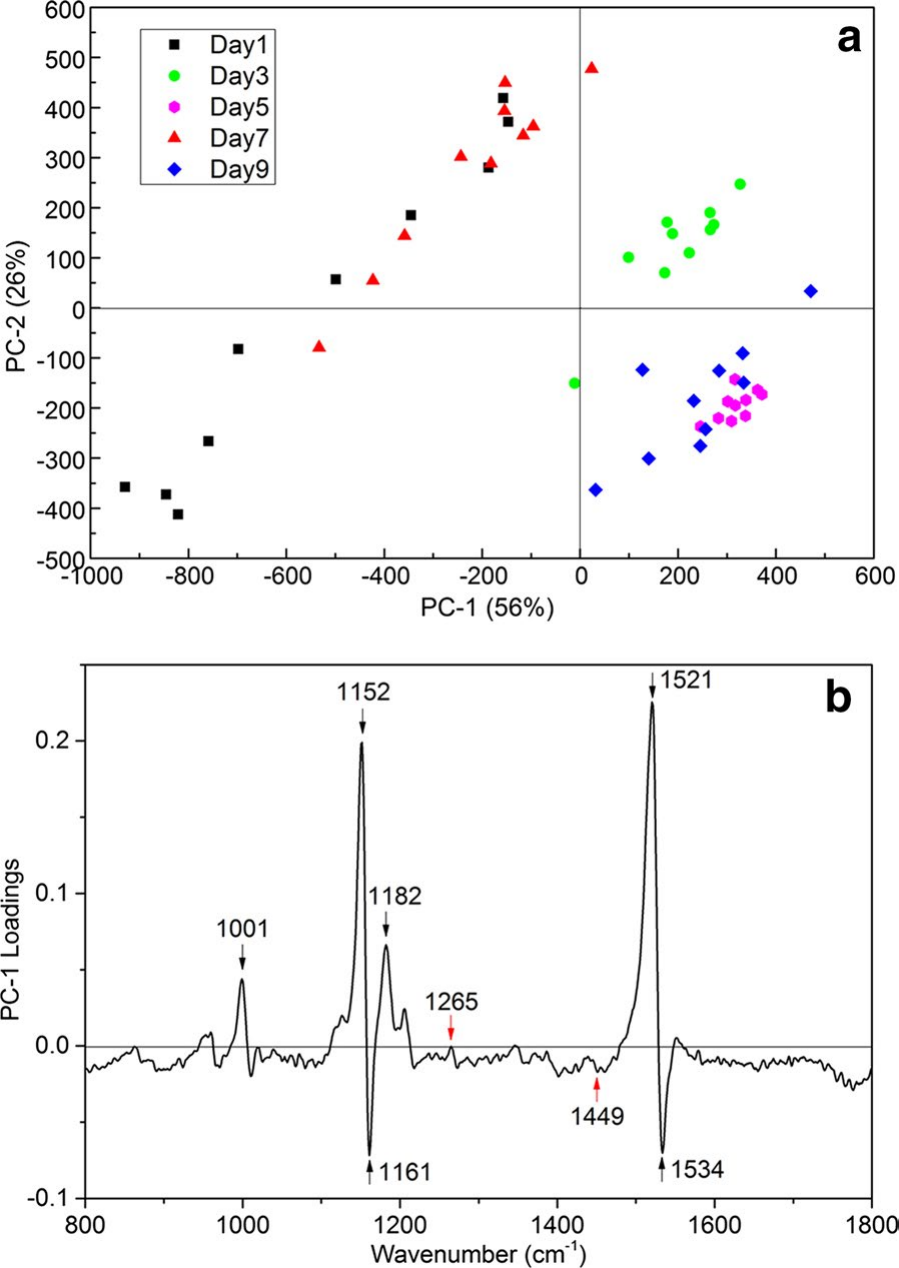
**a** Score plot of PC-1 and PC-2. **b** Corresponding loading plot of PC-1

Raman single data were divided into modeling set and predicting set with a ratio of 2:1. Using PCA-LDA algorithm to model the data of modeling set to discriminate different days of nitrogen stress. The Raman spectra of the cells in different culture days showed obvious clustering in PC-1 (56%) and PC-2 (26%) directions (Fig. 4a). Figure 4b shows the loading plot of PC-1, and shows the contribution of each spectral band of PC-1. The first two PCs described most of chemical information of the Raman spectra of *S. obliquus*. In the PC-1 loading plot, the characteristic peaks with greater contribution rate were basically attributed to β-carotene (1001, 1152, 1161, 1521 cm^−1^) and chlorophyll a (1182, 1534 cm^−1^), which means that these two substances play a major role in the identification of the data in this experiment. During the process of nitrogen stress culture, the contents of β-carotene and chlorophyll a in the microalgae cells in the stable stage were changed greatly.

PCA-LDA discriminant models were established based on the Raman characteristic band of typical chemical substances of *S. obliquus* cells. Four different modeling schemes were used: full band modeling (*F*), characteristic band of pigments (β-carotene, chlorophyll a) modeling (*P*—965, 1007, 1156, 1189, 1526 cm^−1^), characteristic band of lipids modeling (*L*—1266, 1302, 1445, 1656, 1742 cm^−1^), and mixed characteristic band modeling (PL—1007, 1156, 1266, 1445, 1526 cm^−1^). The prediction results (Table 2) of these four models were analyzed. The prediction accuracy of model *F*, *P*, and PL can reach 100%, and model *L* was relatively low, from high to low, was *F* > *P* > PL > *L*. The accuracy of predicting set was slightly lower than of modeling set. These results indicated that the model constructed by Raman spectrum of full band or pigment characteristic band can discriminate *S. obliquus* cells under different nitrogen stress culture times effectively, but accuracy of the model based on lipid characteristic band was relatively low. On this basis, we combined partial Raman bands of pigment and lipid to model (modeling scheme PL) and obtained better results, which means that this prediction method was effective.

**Table 2:** The prediction accuracy of PCA-LDA models

Time	Modeling schemes^a^							
	*F*		*P*		*L*		PL	
	Modeling (%)	Prediction (%)	Modeling (%)	Prediction (%)	Modeling (%)	Prediction (%)	Modeling (%)	Prediction (%)
d1	100	100	100	100	80	40	100	100
d3	100	100	100	100	100	40	100	100
d5	100	100	100	80	90	100	100	100
d7	100	100	100	100	80	60	100	80
d9	100	80	100	80	60	60	100	60
Total	100	96	100	92	82	60	100	88

^a^The modeling scheme is defined based on the Raman characteristic bands used to build the model: *F*—full band modeling; *P*—characteristic band of pigment (β-carotene, chlorophyll a) modeling (965, 1007, 1156, 1189, 1526 cm^−1^); *L*—characteristic band of lipid modeling (1266, 1302, 1445, 1656, 1742 cm^−1^); PL—partial characteristic band of pigment (1007, 1156, 1526 cm^−1^) and partial characteristic band of lipid (1266, 1445 cm^−1^) modeling

The response of lipid characteristic peak was significantly lower than that of pigment characteristic peak, which directly resulted in the low discriminant accuracy of modeling scheme L. The prediction accuracy of modeling scheme P can reach 100%, which indicated that the nitrogen deficiency caused the significant change of pigments in *S. obliquus* cells of stable stage. Although the main purpose of nitrogen stress in this experiment was to induce the accumulation of intracellular lipids, this growth condition also affected the pigments in cells. The effect of pigment change on the model establishment was significant, and the discriminant accuracy of modeling using pigment characteristic bands was better than that of lipid characteristic bands.

### Correlation of Raman mapping and gravimetric method for lipid determination

**Fig. 5:**
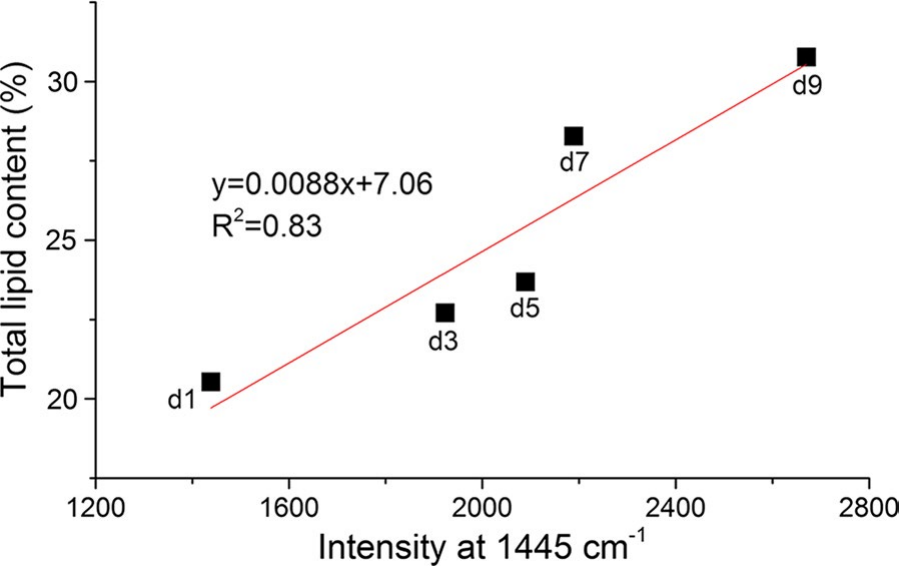
Relationships between total lipid contents and Raman intensity at 1445 cm^−1^ (lipid)

The above results show that Raman spectroscopy is effective for the detection of intracellular lipid in *S. obliquus*. Correlation analysis of total lipid contents measured by gravimetric method and Raman intensity of characteristic peak of lipid (1445 cm^−1^) was carried out (Fig. 5). There was significant correlation (*R* ^2^ = 0.83), indicating that Raman spectroscopy can be applied to the semi-quantitative detection of microalgal lipid content.

## Conclusions

Under the nitrogen stress, significant accumulation of lipid was observed in *S. obliquus* cells. This microalgae species has the ability to synthesize large quantities of long-chain fatty acids and can be used as potential algae for biodiesel production. UFA synthesized by these algae has nutrient value; OCFA has industrial and health care value. The accumulation of lipid in *S. obliquus* was observed by Nile red staining: light yellow spots appeared in cells over time, which were the lipid particles produced by *S. obliquus*.

Raman mapping data of algae cells were visualized. The pyrenoid and intracellular lipid could be located in the pseudo color graph. Raman response high value was dotted in single algae cells, corresponding to the accumulation of lipid particles in the cells. The distribution of SFA and UFA was slightly different. Lipid characteristic peaks were obvious in Raman spectrum, but pigment peaks were more significant.

The Raman spectra of the cells in different culture days showed obvious clustering in PC-1 (56%) and PC-2 (26%) directions. In the PC-1 loading plot, the characteristic peaks with greater contribution rate were basically attributed to β-carotene and chlorophyll a. During the process of nitrogen stress culture, the contents of β-carotene and chlorophyll a in the microalgae cells in the stable stage were changed greatly. Four different models (*F*—full band, *P*—characteristic band of pigments, *L*—characteristic band of lipids, PL—mixed characteristic band) were build and analyzed: the model constructed by Raman spectrum of full band or pigment characteristic band can discriminate *S. obliquus* cells under different nitrogen stress culture times effectively, but accuracy of the model based on lipid characteristic band was relatively low. On this basis, we combined partial Raman bands of pigment and lipid to model (modeling scheme PL) and obtained better results, which means that this prediction method was effective.

Result of correlation analysis of total lipid contents measured by gravimetric method and Raman intensity of characteristic peak of lipid (1445 cm^−1^) showed that there was significant correlation (*R* ^2^ = 0.83), indicating that Raman spectroscopy can be applied to the semi-quantitative detection of microalgal lipid content.

From the above experimental results, it is possible to apply Raman spectroscopy to identify different algal cells with different nitrogen stress days and analyze the intracellular lipid content of algae qualitatively and quantitatively.
